# Supplementary material for: RegBR: A novel Brazilian government framework to classify and analyze industry-specific regulations
Source: PLoS One. 2022 Sep 28;17(9):e0275282. doi: 10.1371/journal.pone.0275282 (PMC9518867; doi:10.1371/journal.pone.0275282)
Supplement: S1 Table — (PDF) [file pone.0275282.s001.pdf]

**Section**	**Column**
1) Agriculture, livestock, forest production, fisheries and aquaculture	1.1) Agriculture, livestock and related services
	1.2) Forest production
	1.3) Fisheries and aquaculture
2) Extractive industry	2.1) Extraction of mineral coal
	2.2) Extraction of oil and natural gas
	2.3) Extraction of metallic minerals
	2.4) Extraction of non-metallic minerals
	2.5) Support activities for mineral extraction
	3.1) Manufacture of food products
	3.2) Beverage manufacturing
	3.3) Manufacture of tobacco products
	3.4) Manufacture of textile products
	3.5) Manufacture of garments and accessories
	3.6) Preparation of leathers and manufacture of leather goods, travel goods and footwear
	3.7) Manufacture of wood products
	3.8) Manufacture of cellulose, paper and paper products
	3.9) Printing and reproduction of recordings
	3.10) Manufacture of coke, petroleum products and biofuels
	3.11) Manufacture of chemical products
	3.12) Manufacture of pharmochemical and pharmaceutical products
	3.13) Manufacture of rubber and plastic products
	3.14) Manufacture of non-metallic mineral products
	3.15) Metallurgy
	3.16) Manufacture of metal products, except machinery and equipment

3) Transformation industry	3.17) Manufacture of computer equipment, electronic and optical products<br>3.18) Manufacture of electrical machinery, apparatus and materials<br>3.19) Manufacture of machinery and equipment<br>3.20) Manufacture of motor vehicles, trailers and bodies<br>3.21) Manufacture of other transport equipment, except motor vehicles<br>3.22) Furniture manufacturing<br>3.23) Manufacture of various products<br>3.24) Maintenance, repair and installation of machinery and equipment
4) Electricity and gas	4.1) Electricity, gas and other utilities
5) Water, sewage, waste and decontamination activities	5.1) Water collection, treatment and distribution<br>5.2) Sewage and related activities<br>5.3) Collection, treatment and disposal of waste; materials recovery<br>5.4) Decontamination and other waste management services
6) Construction	6.1) Construction of buildings<br>6.2) Infrastructure works<br>6.3) Specialized construction services
7) Commerce, Accommodation and Food and Real Estate Services	7.1) Trade and repair of motor vehicles and motorcycles<br>7.2) Wholesale trade, except motor vehicles and motorcycles<br>7.3) Retail trade<br>7.4) Accommodation<br>7.5) Feeding<br>7.6) Real estate activities

8) Transportion, storage and mail	8.1) Land transportation<br>8.2) Water transportation<br>8.3) Air transportation<br>8.4) Storage and auxiliary transport activities<br>8.5) Courier and other delivery activities
9) Information and communication	9.1) Editing and editing integrated with printing<br>9.2) Cinematographic activities, production of videos and television programs; sound recording and music editing<br>9.3) Radio and television activities<br>9.4) Telecommunications<br>9.5) Activities of information technology services<br>9.6) Information service provision activities
10) Financial, insurance and related services	10.1) Financial services activities<br>10.2) Insurance, reinsurance, private pension and health plan<br>10.3) Auxiliary activities of financial services, insurance, private pension and health plans
11) Professional, scientific and technical activities	11.1) Legal, accounting and auditing activities<br>11.2) Activities of company headquarters and business management consultancy<br>11.3) Architectural and engineering services; technical testing and analysis<br>11.4) Scientific research and development<br>11.5) Advertising and market research<br>11.6) Other professional, scientific and technical activities<br>11.7) Veterinary activities
	12.1) Non-real estate rentals and management of non-financial intangible assets

12) Administrative activities and complementary services	12.2) Selection, agency and hiring of labor<br>12.3) Travel agencies, tour operators and reservation services<br>12.4) Surveillance, security and investigation activities<br>12.5) Services for buildings and landscape activities<br>12.6) Office, administrative support and other services provided mainly to companies
13) Public administration, defense and social security	13.1) Public administration, defense and social security
14) Education	14.1) Education
15) Human health and social service	15.1) Human health care activities<br>15.2) Human health care activities integrated with social assistance, provided in collective and private residences<br>15.3) Social assistance services without accommodation
16) Arts, culture, sports and recreation	16.1) Artistic, creative and show activities<br>16.2) Activities related to cultural and environmental heritage<br>16.3) Gambling and betting activities<br>16.4) Sports and recreation and leisure activities
17) Other services	17.1) Activities of membership organizations<br>17.2) Repair and maintenance of computer and communication equipment and personal and domestic objects<br>17.3) Other personal service activities<br>17.4) Domestic services<br>17.5) International organizations and other extraterritorial institutions

18) Non-regulatory	18.1) Budget<br>18.2) Public administration<br>18.3) Normative
