# Supplementary material for: RegBR: A novel Brazilian government framework to classify and analyze industry-specific regulations
Source: PLoS One. 2022 Sep 28;17(9):e0275282. doi: 10.1371/journal.pone.0275282 (PMC9518867; doi:10.1371/journal.pone.0275282)
Supplement: Attachment — Submitted filename: Response to Reviewers .pdf [file pone.0275282.s003.pdf]

Dear Prof. Sathishkumar V E,

On behalf of all the authors of the manuscript "RegBR: A novel Brazilian government framework to classify and analyze industry-specific regulations," we would like to thank you very much for revising our manuscript and for the relevant comments provided by you and the two reviewers, which helped us to improve our work. We submit our revised manuscript for appreciation and also a point-by-point response to the reviewer's comments.

As suggested in the decision e-mail, we also decided to modify the funding statement in order to suit the journal's requirements. The new funding statement is as follows:

This research was supported by the National School of Public Administration – ENAP, Government of Brazil (Grant TED UnB-ENAP 83/2018). There was no additional external funding received for this study.

For the acknowledgements section of our manuscript, we also like to slightly modify the statement to the following one:

The authors wish to thank the National School of Public Administration (ENAP) for all the support and partnership in the development of the RegBR project. We also would like to thank the relevant feedback received by the regulatory agencies, which contributed with clear ideas so that the project could be used in practice by policy makers.

We hope that the improvements we have made to this paper will be enough to enable it to be considered for publication by this renowned journal.

Thank you for considering our manuscript.

Best regards,

The authors

Academic editor:

1. Please ensure that your manuscript meets PLOS ONE's style requirements, including those for file naming. The PLOS ONE style templates can be found at

https://journals.plos.org/plosone/s/file?id=wjVg/PLOSOne_formatting_sample_main_body.pdf and https://journals.plos.org/plosone/s/file?id=ba62/PLOSOne_formatting_sample_title_authors_affiliations.pdf

R. We have made changes to figures, tables, and author affiliations formatting and we now confirm that our manuscript meets PLOS ONE's style requirements. We used PACE digital diagnostic tool to ensure that our Figures meet PLOS requirements.

2 Thank you for stating in your Funding Statement:

"This research was partially supported by the National School of Public Administration – ENAP, Government of Brazil (Grant TED UnB-ENAP 83/2018)."

Please provide an amended statement that declares *all* the funding or sources of support (whether external or internal to your organization) received during this study, as detailed online in our guide for authors at http://journals.plos.org/plosone/s/submit-now. Please also include the statement "There was no additional external funding received for this study." in your updated Funding Statement.

Please include your amended Funding Statement within your cover letter. We will change the online submission form on your behalf.

R. We would like to change our funding statement to the following:

"This research was supported by the National School of Public Administration – ENAP, Government of Brazil (Grant TED UnB-ENAP 83/2018). There was no additional external funding received for this study."

3. Thank you for stating the following in the Acknowledgments Section of your manuscript:

"The authors wish to thank the National School of Public Administration (ENAP) for all the support and partnership within the development of the RegBR project, providing funding, infrastructure and establishing contact with regulatory agencies. We also would like to thank the relevant feedback received by the Brazilian regulatory agencies, who contributed with clear ideas so that the project could be used in practice by police makers"

We note that you have provided additional information within the Acknowledgements Section that is not currently declared in your Funding Statement. Please note that funding information should not appear in the Acknowledgments section or other areas of your manuscript. We will only publish funding information present in the Funding Statement section of the online submission form.
Please remove any funding-related text from the manuscript and let us know how you would like to update your Funding Statement. Currently, your Funding Statement reads as follows:

"This research was partially supported by the National School of Public Administration – ENAP, Government of Brazil (Grant TED UnB-ENAP 83/2018)."

Please include your amended statements within your cover letter; we will change the online submission form on your behalf.

R. We also would like to change our Acknowledgments section to the following:

The authors wish to thank the National School of Public Administration (ENAP) for all the support and partnership in the development of the RegBR project. We also would like to thank the relevant feedback received by the regulatory agencies, which contributed with clear ideas so that the project could be used in practice by policy makers.

4. Please review your reference list to ensure that it is complete and correct. If you have cited papers that have been retracted, please include the rationale for doing so in the manuscript text, or remove these references and replace them with relevant current references. Any changes to the reference list should be mentioned in the rebuttal letter that accompanies your revised manuscript. If you need to cite a retracted article, indicate the article's retracted status in the References list and also include a citation and full reference for the retraction notice.

R. We have confirmed that our reference list is complete and correct.

Reviewers' comments:

- Reviewer #3

  In this paper, the authors apply machine learning, deep learning and transfer learning models along with word weighting and embedding schemes to generate framework to classify industry specific regulations. The various combinations of techniques used by the authors are appreciable. The paper is well organized and written. The interpretation and description of the experimental results are also explained clearly. However, this manuscript have some weak points, it should be further improved before consider for publication. Some of my observations are

  R. The modifications associated with reviewers' #3 comments are highlighted in green in the *Revised Manuscript with Track Changes* document. Next, we provide a point-by-point response to the reviewers' #3 comments.

1. Figures 1,2,3,7 need to be drawn clearly and explanation about each figure to be incorporated in corresponding section.

   R. Thank you for this comment. We have redrawn the indicated Figures and added a clearer explanation in the corresponding sections. In order to adapt our work to the journal's style format, some tables have become figures and that's why the new figures in this comment are now the Figures 2, 3, 4 and 8.

2. In figure 5, x-axis legend is not mentioned and proper explanation is required.

   R. Thank you for noticing that. We adjusted the image adding the x-axis legend and its scale. Also, we added a paragraph explaining the image and an example of its interpretation. Fig 5 is now named as Fig 6.

3. In figure 9, only 8 classes are displayed? What about other classes. Justification is required.

   R. We appreciate the question. In response, we only displayed the classes that presented a significant correlation level between their KPCA projection and their complexity metrics. A total of 8 classes satisfied these conditions. We made it clearer in the manuscript, at the subsection *Linguistic complexity* right before Fig 9.

4. Why validation is not performed? Justify

   R. Thank you for bringing up this point. In fact, RegBR is the first Brazilian regulatory framework that creates centralized regulatory metrics, and for this reason, there is no similar work to be compared with. On the other hand, we can correlate the observed results with known episodes of regulatory change in Brazilian history, such as the time of the creation of the regulatory agencies, the implementation of new economic plans or the energy crisis that happened in the 2000s, as related in the *Regulatory metrics conception* subsection. Also, after presenting the results to the national regulatory agencies, we received feedback in the sense that our metrics results corroborated with internal metrics created by the agencies. An example of this fact is that the national water agency (ANA), which uses a plain language approach to monitor the linguistic complexity of their texts, informs us that they were expecting ANA's agency to have one of the biggest linguistic complexity within the sectors for the types of normative acts analyzed in our work, which was confirmed by our linguistic complexity metric.

5. Why the authors specifically choose CNN and LSTM classifiers?

   R. Thank you for the question. The reasons for choosing CNN and LSTM are based on two main factors. First, they are often used in many NLP applications. Secondly, before the creation of the Transformer architecture, they were used as building blocks for some state-of-the-art NLP models. These two points led us to include models based on these two neural network designs to our benchmark. We added a paragraph in the manuscript to make that choice clearer.

6. According to the results obtained, if statistical models perform better than machine, deep and transfer learning models, then what is the use of these latest techniques for analysis? Is hypertuning of these models not done properly? Justification is required.

   R. Thank you for raising this point. We added more information in the text regarding the importance of benchmarking different models. For some NLP tasks, such as translation, there are standard datasets that are used to evaluate models. In that case, comparing the proposed model with the current state-of-the-art is enough most of the time. However, in our application, there were no past performances to serve as a baseline to indicate what would be considered a "good" model. Thus, we performed an evaluation of different models and approaches. With regard to the hypertuning, we

used grid-search with the values for the parameters that are described in the literature and we have updated this information in the manuscript. Moreover, in the text, we raised some possible reasons why the transfer-learning methods presented underwhelming results.

Once again, we thank the reviewer for devoting his/her time to assess our manuscript and for the helpful comments which have guided us to enrich the original manuscript. We hope that our manuscript and revision of the manuscript are satisfactory.

- Reviewer #4

R. The modifications associated with reviewers' 4 comments are highlighted in orange in the *Revised Manuscript with Track Changes* document. Next, we provide a point-by-point response to the reviewers' 4 comments.

1. Manuscript needs English language check as the sentence formation errors occur throughout the manuscript

R. We performed a manuscript language review and corrected grammar and sentence formation errors. We hope that this revised version of the manuscript will meet the language requirements.

2. What strategy is used to select hyperparameters?

R. Thank you for your question. We made it clearer in the paper how we select the hyperparameters. We performed a grid-search using the hyperparameter values often used in the literature for this kind of task.

3. Explain the dataset used in detail. Make a table to summarize the dataset features.

R. Thank you for the comments. In the text classification task, all the features were derived from the normative act texts themselves. To summarize the data workflow, first we build an ETL (Extract, transform, load) system to gather normative act texts from various sources and centralize them in a database. Then, we prepared the data by removing stop words, malformed characters and numbers, and standard sentences and headers that appear in all normative texts. Lastly, we transform text to numeral representation in different ways depending on the model used, like using tf-idf, word embeddings, or in the case of transformer-based models, the specific encoder proposed by the model architecture.

More details on each model's feature extraction approach are available in the subsection *Details on Text Classification*.

4. Explain the proposed work in detail

R. Our paper proposes a novel regulatory framework applicable to Brazil with the goal of producing relevant information on the national regulatory situation in order to: promote active transparency of public information; subsidize new regulatory studies based on RegBR data; and assist decision makers to measure what their organization produces in terms of volume and characteristics of regulations. For the first time, we classify normative acts in industry-specific classes and create a

set of new regulatory metrics that concern linguistic complexity, restrictiveness, legal interest, and industry-specific citation relevance. We made some adjustments in the manuscript such that the goals and applications of the proposed work are better explained.

5. Elaborate Normative acts.

   R. By a dictionary definition, a *normative act* means any law, decree, resolution, regulation, administrative direction, instruction, rule, ordinance, or other decision that creates legal consequences for more than one individual. We also added this definition in the manuscript, in the *Normative Acts data structure* subsection.

6. Application of the proposed system should be explained in detail.

   R. Thank you for raising this point. We are producing a second paper focused exclusively on RegBR applications, but in order to make the applications clear in this article, we added a new paragraph in the *Introduction* section explaining in more detail the possible applications of the system.

7. What is the need of using ensemble classifier?

   R. Ensemble models tend to yield better results when there is a significant diversity among the models. In our case, we combine a ridge classifier, which is a classifier that converts the task into a regression and generates labels accordingly, and an SVM classifier. These two models have a few complementary strengths and weaknesses, and when combined, generate a more precise and robust prediction.

8. Whole manuscript should be reorganised following standard scientific article requirements

   R. Parts of the manuscript text, figures, and tables have been rewritten so we could meet all PLOS ONE organization and style requirements available in https://journals.plos.org/plosone/s/file?id=wjVg/PLOSOne_formatting_sample_main_body.pdf. We hope that the improvements we have made to this paper should be enough to enable it to be published by this renowned journal. We remain open to modifying specific parts of the manuscript that you feel don't meet the journal requirements.

We would like to thank the reviewer for providing some helpful comments and suggestions that have improved our manuscript. We hope that our responses and revisions of the manuscript are satisfactory.
