# Supplementary material for: RegBR: A novel Brazilian government framework to classify and analyze industry-specific regulations
Source: PLoS One. 2022 Sep 28;17(9):e0275282. doi: 10.1371/journal.pone.0275282 (PMC9518867; doi:10.1371/journal.pone.0275282)
Supplement: Attachment — Submitted filename: Plos one-review comments-07.08.22.docx [file pone.0275282.s002.docx]

RegBR: A novel Brazilian government framework to classify and analyze industry specific Regulations

Reviewer Comments:

In this paper, the authors apply machine learning, deep learning and transfer learning models along with word weighting and embedding schemes to generate framework to classify industry specific regulations. The various combinations of techniques used by the authors are appreciable. The paper is well organized and written. The interpretation and description of the experimental results are also explained clearly. However, this manuscript have some weak points, it should be further improved before consider for publication. Some of my observations are

1. Figures 1,2,3,7 need to be drawn clearly and explanation about each figure to be incorporated in corresponding section.

2. In figure 5, x-axis legend is not mentioned and proper explanation is required.

3. In figure 9, only 8 classes are displayed? What about other classes. Justification is required.

4. Why validation is not performed? Justify

5. Why the authors specifically choose CNN and LSTM classifiers?

6. According to the results obtained, if statistical models perform better than machine, deep and transfer learning models, then what is the use of these latest techniques for analysis? Is hypertuning of these models not done properly? Justification is required.
